# Heat-shock protein 90α is involved in maintaining the stability of VP16 and VP16-mediated transactivation of α genes from herpes simplex virus-1

**DOI:** 10.1186/s10020-018-0066-x

**Published:** 2018-12-22

**Authors:** Yiliang Wang, Rongze Wang, Feng Li, Yun Wang, Zhen Zhang, Qiaoli Wang, Zhe Ren, Fujun Jin, Kaio Kitazato, Yifei Wang

**Affiliations:** 1 Guangzhou Jinan Biomedicine Research and Development Center, Institute of Biomedicine, College of Life Science and Technology, Jinan University, Guangzhou, China; 2 Key Laboratory of Virology of Guangzhou, Jinan University, Guangzhou, China; 3 Key Laboratory of Bioengineering Medicine of Guangdong Province, Jinan University, Guangzhou, China; 4 College of Pharmacy, Jinan University, Guangzhou, China; 5 Key Laboratory for Major Obstetric Diseases of Guangdong Province, Department of Obstetrics and Gynecology, Third Affiliated Hospital of Guangzhou Medical University, Guangzhou, China; 6 Integrated Chinese and Western Medicine Postdoctoral Research Station, Jinan University, Guangzhou, China; 7 Division of Molecular Pharmacology of Infectious Agents, Department of Molecular Microbiology and Immunology, Graduate School of Biomedical Sciences, Nagasaki University, 1-14 Bunkyo-machi, Nagasaki, 852-8521 Japan

**Keywords:** Herpes simplex virus-1, Heat-shock protein 90α, Autophagy, α genes, VP16

## Abstract

**Background:**

Numerous host cellular factors are exploited by viruses to facilitate infection. Our previous studies and those of others have shown heat-shock protein 90 (Hsp90), a cellular molecular chaperone, is involved in herpes simplex virus (HSV)-1 infection. However, the function of the dominant Hsp90 isoform and the relationship between Hsp90 and HSV-1 α genes remain unclear.

**Methods and results:**

Hsp90α knockdown or inhibition significantly inhibited the promoter activity of HSV-1 α genes and downregulated virion protein 16(VP16) expression from virus and plasmids. The Hsp90α knockdown-induced suppression of α genes promoter activity and downregulation of α genes was reversed by VP16 overexpression, indicating that Hsp90α is involved in VP16-mediated transcription of HSV-1 α genes. Co-immunoprecipitation experiments indicated that VP16 interacted with Hsp90α through the conserved core domain within VP16. Based on using autophagy inhibitors and the presence of Hsp90 inhibitors in ATG7^−/−^ (autophagy-deficient) cells, Hsp90 inhibition-induced degradation of VP16 is dependent on macroautophagy-mediated degradation but not chaperone-mediated autophagy (CMA) pathway. In vivo studies demonstrated that treatment with gels containing Hsp90 inhibitor effectively reduced the level of VP16 and α genes, which may contribute to the amelioration of the skin lesions in an HSV-1 infection mediated zosteriform model.

**Conclusion:**

Our study provides new insights into the mechanisms by which Hsp90α facilitates the transactivation of HSV-1 α genes and viral infection, and highlights the importance of developing selective inhibitors targeting the interaction between Hsp90α and VP16 to reduce toxicity, a major challenge in the clinical use of Hsp90 inhibitors.

**Electronic supplementary material:**

The online version of this article (10.1186/s10020-018-0066-x) contains supplementary material, which is available to authorized users.

## Background

Herpes simplex virus (HSV) type 1 (HSV-1) infection causes major global health challenges, particularly HSV encephalitis (Roehm et al., 2016). The long-term application of conventional antiviral drugs, such as acyclic purine nucleoside analogues, against herpesvirus is the leading cause of frequent emergence of drug resistant virus; thus, novel therapeutic targets are urgently needed (Clercq & Herdewijn, 2002; Coen & Schaffer, 2003). Given that viruses are obligate parasites, several host factors utilized by virus are promising therapeutic targets (Org et al., 2017). Heat-shock protein 90 (Hsp90) is such a promising therapeutic host target against viral infection and has attracted intensive attention in the development of novel antiviral drugs because of its functions in the life cycle of multiple viruses, including human immunodeficiency virus (HIV)-1, enterovirus 71 (EV71), HSV-1, and influenza virus (Wang et al., 2017).

Hsp90 is a conserved molecular chaperone and facilitates the maturation of a wide range of proteins and participates in the trafficking of client proteins within the cellular milieu (Tatokoro et al., 2015). Our previous studies have demonstrated that Hsp90 inhibitors, including 17-AAG, SNX-2112, and AT533, exhibit strong antiviral activity against HSV-1 in vitro and in vivo; in particular, AT533 shows excellent antiviral activity in a herpes simplex keratitis (HSK) rabbit model, with greater efficiency than that of acyclovir (ACV) (Wang et al., 2017; Xiang et al., 2012a; Zhong et al., 2014; Wang et al., 2018; Li et al., n.d.; Ju et al., 2011). Hsp90 inhibition suppresses the nuclear trafficking of HSV-1 capsid proteins in vitro (Zhong et al., 2014) and leads to mislocalization of the viral DNA polymerase, further limiting HSV-1 infection (Burch & Weller, 2005).

However, the functional differences between different isoforms of Hsp90 during HSV-1 infection are unclear. Indeed, Hsp90 has four major isoforms, Hsp90α, Hsp90β, tumor necrosis factor receptor-associated protein 1, and 94-kDa glucose-regulated protein (GRP94) (Whitesell & Lindquist, 2005). Of the four Hsp90 isoforms, two are cytoplasmic, the stress-induced Hsp90α and constitutively expressed Hsp90β. The majority of viruses, such as EV71, HIV-1, and hepatitis B virus (HBV), utilize these two isoforms (Burch & Weller, 2005; Whitesell & Lindquist, 2005; Wang et al., 2013; Reyes-Del Valle et al., 2005; Voss et al., 2000). In contrast, few studies have focused on the roles of the other isoforms in viral infection except for GRP94. GRP94 can be utilized by hepatitis C virus to inhibit the host immune system response and block viral-induced apoptosis (Song et al., 2008).

Previous studies have failed to exclude the possibility that Hsp90 is involved in the transcription of HSV-1 α genes. Hsp90 modulates the transcription of several genes at multiple levels and our earlier study demonstrated that Hsp90 inhibition reduces α genes expression (Zhong et al., 2014; Zhao et al., 2005; Wan & Lenardo, 2010; Floer et al., 2008; Khurana & Bhattacharyya, 2015). Therefore, an association may exist between Hsp90 and the expression of HSV-1 α genes. Indeed, Hsp90 is required by several viruses, such as HBV, where it does facilitate RT-ε interaction and initiate reverse transcription (Wang et al., 2017; Hu et al., 2004). In the HSV-1 life cycle, three kinetic classes of genes are sequentially expressed, namely the immediate-early (IE) α genes, early (E) β genes, and late (L) γ genes. The virus gene expression is associated with the comprehensive modulation of host and viral factors (Weir, 2001). In the IE phase, virion protein 16 (VP16) is a crucial tegument protein involved in the assembly of a transactivation complex containing octamer-binding transcription factor 1 (Oct-1) and host cell factor 1 (HCF-1) that binds to the promoters of α genes, including *α0* and *α4 (Wysocka & Herr,* 2003*;* *Amici et al.,* 2006*)*. When the complex loads into the corresponding site, it then recruits several transcription factors, including RNA polymerase and chromatin-remodeling enzymes, and the Set1 histone methyl transferase is recruited, which removes the heterochromatic H3K9 methylation (Liang et al., 2009; Narayanan et al., 2007). Subsequently, α proteins are induced and engage in the modulation of β and γ genes transcription and the feedback regulation of α genes (Deluca & Schaffer, 1985; Jugovic et al., 1998; Lin et al., 2010; Sekulovich et al., 1988). Several novel host factors are found to be sequentially utilized by HSV-1 to accomplish the IE phase. Nuclear lamina A, a major component of the nuclear lamina, is crucial for nuclear localization and the formation and assembly of the VP16 activator complex (Silva et al., 2012). In addition, the super elongation complex (SEC) modulates the transcription of α genes by forming the SEC-P-TEFb complex, which is associated with transcriptional elongation (Alfonso-Dunn et al., 2017). Collectively, host factors, including Hsp90, are used by the virus to facilitate the transcription of viral genes. However, the regulation of HSV-1 α genes involving Hsp90 remains to be elucidated.

Accordingly, in this study, we evaluated the role of specific Hsp90 isoforms in regulating HSV-1 α genes and explored the underlying molecular mechanism, particularly in regards to VP16. In addition, the relationship between Hsp90 and HSV-1 α genes was also confirmed in an HSV-1 infection-mediated zosteriform mouse model by assessing the efficacy of Hsp90 inhibitor-containing gels against HSV-1 infection.

## Materials and methods

### Cells and viruses

Vero cells (ATCC CCL81) were cultured as described previously (Zhong et al., 2014). SH-SY5Y cells (ATCC CRL-2266) were cultured at 37 °C in a humid atmosphere with 5% CO_2_. HEK 293 T cells (ATCC CRL-11268) were grown and maintained at 37 °C in a humidified incubator. MEF WT and autophagy-deficient ATG7^−/−^ cells were obtained and cultured as indicated in our previous study (Liu et al., 2016). All experiments were performed with HSV-1 strain 17 (ATCC, VR733), which was obtained and stored as previously indicated (Zhong et al., 2014; Xiang et al., 2012b; Zheng et al., 2014).

### Inhibitors, antibodies, siRNAs, and plasmids

The inhibitor 17AAG was purchased from Sigma-Aldrich (St. Louis, MO, USA). SNX-2112 and SNX-25a (AT533) were synthesized according to a previously described procedure (Xiang et al., 2012a; Jin et al., 2009). MG132 (S2619) was purchased from Selleck (Houston, TX, USA), and CQ (C6628), 3-MA (M9281), and CHX (C7698) were purchased from Sigma-Aldrich. All of the inhibitors were used at noncytotoxic concentrations. The cytotoxicities of drugs, siRNAs, and transient expression were monitored by MTT (M2128) assays.

Anti-VP16 (ab11026), anti-Hsp90α (ab59459), anti-gB (ab6506),anti-ICP5 (ab6508) and anti-p62(ab56416) antibodies were purchased from Abcam (Cambridge, UK). Anti-glyceraldehyde 3-phosphate dehydrogenase (GAPDH; 2118), anti-Hsp90β (5087S), anti-ATG7 (8558), anti-ubiquitin (#3936), and anti-p21 (2947) antibodies were purchased from Cell Signaling Technology (Danvers, MA, USA). Anti-LC3B (L7543) and anti-FLAG (M2) (1804) antibodies were purchased from Sigma-Aldrich. The anti-Oct-1 (sc-8024) antibody was obtained from Santa Cruz Biotechnology (Santa Cruz, CA, USA) and the Alexa Fluor 488/594-conjugated goat anti-mouse and anti-rabbit IgG were purchased from Invitrogen (Carlsbad, CA, USA). All the eukaryotic expression plasmids, including pcDNA3.1(+)-VP16, pcDNA3.1(+)-Hsp90α, p3xFLAG-VP16, and pCMV-HA-Hsp90α, were generated in our laboratory. cDNA was generated using RNA obtained from HSV-1-infected cells as a template to amplify the VP16 coding sequence. Viral DNA extracted from HSV-1(F)-infected cells was utilized as a template to amplify the promoters of *α0* and *α4*. All constructed plasmids were verified by DNA sequencing (Invitrogen). For information on the primers and vectors used to construct the plasmids, see Additional file 1: Table S1. For siRNA sequences, see Additional file 1: Table S2; all siRNAs were purchased from Gene Pharma (Shanghai, China).

### Transfection

For transfection, cells were plated into 6-well plates 1 day before transfection at a density of 10^5^ cells/well or at 50–60% confluency. Transfection of SH-SY5Y cells and Vero cells with the indicated plasmids was performed with Lipofectamine 3000 transfection reagent according to the manufacturer’s instructions (Invitrogen). Briefly, 3 μg of the corresponding plasmids and Lipofectamine 3000 reagent were diluted in 100 μl Opti-MEM I reduced-serum medium (Invitrogen) and the diluted plasmids were then added to the Lipofectamine 3000 (1:1 ratio), mixed, and incubated at room temperature for 10 min. The transfection mixture was then added to cells at 50–60%. For siRNA transfections, Lipofectamine RNAiMax (Invitrogen) reagent was used according to the manufacturer’s instructions. Briefly, siRNAs (100 nM) and Lipofectamine RNAiMAX reagent were diluted with Opti-MEM, mixed thoroughly, and the diluted siRNA was then added to the Lipofectamine RNAiMAX reagent (1:1 ratio), mixed, and incubated at room temperature for 5 min. The mixture was then added to cells (at 50–60% confluence).

### Dual luciferase reporter (DLR) assays

DLR assays were performed using a dual luciferase assay system (Promega, Madison, WI, USA) according to the manufacturer’s instructions. Briefly, cells were transfected with VP16 expression plasmids and reporter plasmids containing the intended promoter (pGL4.12 [luc2p]-*α0* promoter [pGL-*α0*] or pGL4.12 [luc2p]-*α4* promoter [pGL-*α4*]), with the pRL-TK plasmids expressing *Renilla* luciferase as an internal control to normalize the transfection efficiency. When siRNA transfection was required, cells were selectively cotransfected with siRNA against Hsp90α or Hsp90β and the corresponding reporter plasmids mentioned above. We performed the indicated treatments at 24 h post transfection and then detected luciferase activity using a Dual Luciferase Reporter Assay System (E1910) according to the manufacturer’s instructions. Relative luciferase activity (RLA) was determined by normalizing signals to *Renilla* luciferase activity. Each experiment was repeated three times and the means were calculated for statistical analysis.

### Viral titration and viral plaque assay

Viral titration was used to determine cytopathic effects (CPEs) in Vero cells to calculate the 50% tissue culture infectious dose (TCID_50_) (Reed & Muench, 1938). Subsequently, the TCID_50_/mL was converted into plaque-forming units (PFU)/mL. Plaque reduction assays were used to determine the appropriate dilution for plaque assays, as described in our previous study (Pei et al., 2011). Briefly, cells were seeded into 24-well plates at a high density for 24 h and then infected with HSV-1 for 2 h. The overlay medium consisting of maintenance medium containing 1% methylcellulose (SIJIA BIOTECH, Guangzhou, China) in the presence or absence of inhibitors was added to each well. After incubation for 72 h, the cell monolayers were fixed with 10% formalin and stained with 1% crystal violet (Beyotime, Suzhou, China). Plaques were counted, and the percentage of inhibition was calculated. Viral titration of the skin tissue from HSV-1-infected mice was determined as previously indicated with minor revision (Van et al., 2004). Briefly, a 1 cm^2^ piece of skin were removed as detailed below in 2.9 section and then placed in 1 ml of DMEM (Invitrogen). The specimens were repeatedly frozen at − 80 °C for three times then centrifuged at 12,000×*g* for 5 min at 4 °C and the supernatant collected. The supernatant was 10-fold serially diluted and then tested for plaque formation to determine the virus titer in the original tissue sample. The amount of infectious viral particles in the supernatant was determined by standard PFU assays on confluent monolayers of Vero cells.

### Western blotting

Cells were lysed in sodium dodecyl sulfate (SDS) buffer (Beyotime) containing 1 mM phenylmethylsulfonyl fluoride (PMSF) (Beyotime), and the protein concentration was measured using an enhanced bicinchoninic acid (BCA) protein assay kit (Beyotime). The cell lysates were then mixed with the appropriate volume of 5× SDS-polyacrylamide gel electrophoresis (PAGE) buffer (Beyotime) and SDS buffer to obtain the same concentration and then boiled for 10 min. Finally, samples were analyzed by SDS-PAGE on 8–15% gradient gels, transferred to polyvinylidene difluoride membranes (Merck Millipore, Darmstadt, Germany), probed with the indicated primary antibodies, and then incubated with horseradish peroxidase-conjugated secondary antibodies. All proteins of interest were detected by enhanced chemiluminescence (Millipore). The band intensity of each protein was calculated using Quantify One software (Bio-Rad, Hercules, CA, USA) and normalized to that of GAPDH.

### Immunoprecipitation assay

Cells were washed once with phosphate-buffered saline (PBS) and then lysed with ice-cold cell lysis buffer containing 1 mM PMSF on ice for 30 min for immunoprecipitation (Beyotime). Subsequently, to collect soluble lysates, primary cell lysates were centrifuged at 14000×*g* for 10 min at 4 °C. After the protein concentrations of the soluble lysates was determined using a BCA protein assay kit, 1 mg of protein was incubated with the indicated primary antibody at 1 μg for 4 h at 4 °C before being agitated with 40 μl PLUS-Agarose (Santa Cruz Biotechnology) at 4 °C overnight to capture the immune complexes. The immunoprecipitated proteins were further collected by centrifugation at 14000×*g* for 15 s at 4 °C and then washed five times with precooled lysis buffer containing 1 mM PMSF to remove the unbound proteins and prevent the degradation of bound proteins. Immunoprecipitated proteins were separated by adding 40 μl SDS loading buffer and boiling for 5 min and then subjected to western blot analysis with specific primary and secondary antibodies. To minimize the interference from denatured IgG heavy chains on the indicated band, IPkine HRP AffiniPure Goat Anti-Mouse IgG Light Chain (Abbkine, CA, USA) and IPkine HRP AffiniPure Mouse Anti-Rabbit IgG Light Chain (Abbkine) antibodies were used at a dilution of 1: 4000.

### Quantitative real-time polymerase chain reaction (qPCR)

Total RNA was extracted from cells with TRIzol reagent (TIANGEN, Beijing, China) according to the manufacturer’s protocol. A 1 cm^2^ piece of skin extending between the inoculation site and the anterior midline was removed, and the RNA extracted using an RNAprep Pure Tissue Kit (TIANGEN) according to the manufacturer’s instructions. The RNA concentration was measured at 260/280 nm using a NanoPhotometer P330 spectrophotometer (IMPLEN, Munich, Germany), and 1 μg RNA was then reverse transcribed into cDNA using a PrimeScript RT Reagent Kit (TAKARA, Dalian, China). The qPCR assays were performed using a SYBR Premix Ex Taq II Kit (TAKARA), a CFX96 real-time PCR system (Bio-Rad), and 250 nM of each primer (Sango, Shanghai, China) (Xiang et al., 2012a). The mRNA levels were standardized against the housekeeping gene *GAPDH* in SH-SY5Y cells or the *18 s* gene in Vero cells using the 2^−ΔΔCT^ method with the CFX96 system software (Bio-Rad). Gene-specific primer pairs used in the qPCR assay are provided in the Additional file 1: Table S3.

### Zosteriform model of HSV-1 infection in the context of Hsp90 inhibition

The zosteriform model of infection was established as indicated previously and detailed protocols are available (Van et al., 2004; Wang et al., 2016a). Briefly, 10-weeks old female and male C57BL/6 mice (Guangdong Medical Laboratory Animal Center) were randomly divided into the indicated groups. The flank hair of each mouse was clipped and depilated with Ice King skin hair removal cream (Ice King, Shanghai, China). The mice were then allowed to relax for three days while the skin recovered from the hair removal cream stimulation. The mice were lightly anesthetized with an injection of phenobarbital sodium, a 20-μl volume droplet containing 10^5^ PFU HSV-1 was placed on the skin of each mouse near the top of the spleen, corresponding to the tenth thoracic dermatome (T10). The skin was then scarified 20 times with a 27-gauge needle through the droplet and the viral suspension was allowed to dry. Infected mice were observed daily and a gel mixture containing 0.02% or 0.04% AT533 was applied to the mouse skin once per day starting throughout the entire process from 24 h post inoculation with the HSV-1. The control group was treated with gels containing no drugs. The gels containing AT533 were prepared according to our previous study (Xiang et al., 2012a). Mice were sacrificed on day 7 after HSV-1 infection when the abraded infected skins displayed significant skin lesions. The relative sizes of skin lesions were quantified by measuring the width of the zosteriform lesions and used as an indicator of the severity of the skin lesion. A one square centimeters piece of skin extending between the inoculation site and the anterior midline was removed and the total RNA extracted using an RNA prep Pure Tissue Kit (TIANGEN). Total proteins with SDS lysis buffer containing 1 mM PMSF. All specimens were stored in sterile tubes, frozen at − 80 °C, and subjected to analysis within 3 days of collection. All instruments utilized in obtaining the tissue specimens were washed in methylated spirits between each manipulation. All animal experiments were performed with the approval of the Guangzhou (Jinan) Biomedical Research and Development Center.

## Results

### Hsp90α is involved in modulating the promoter activity of HSV-1 α genes

**Fig. 1:**
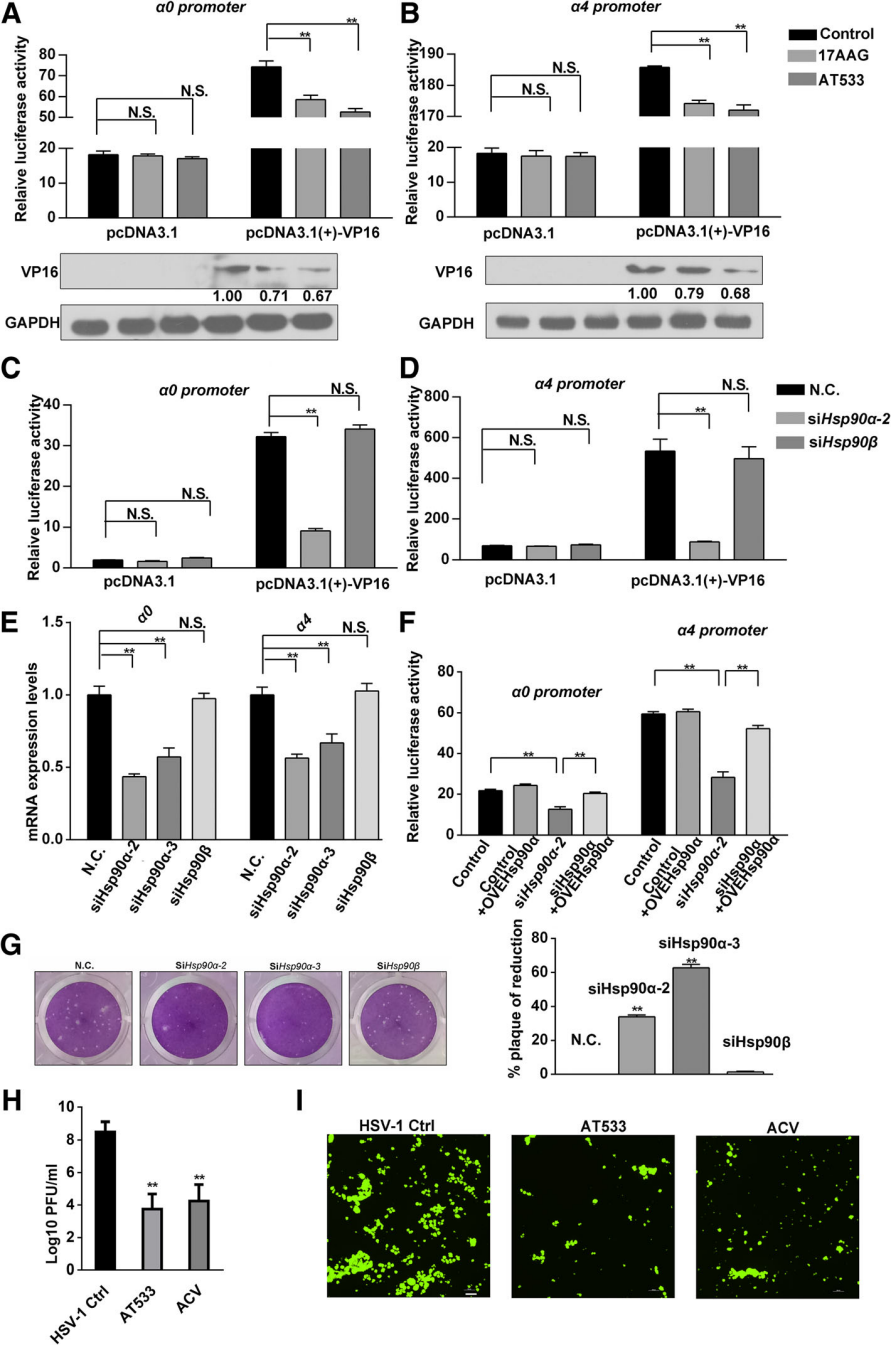
Hsp90α is involved in modulating the transcription of HSV-1 α genes. **a**, **b** The promoter activity of HSV-1 α genes in the presence of Hsp90 inhibitors. Vero cells were cotransfected with reporter plasmids as described in section 2.4 of the Materials and Methods. Cells were then treated with 17AAG (0.5 μM) or AT533 (2 μM) for 2 h. Cell lysates were subjected to luciferase activity assays (**a** and **b**, upper) and analyzed by western blotting to detect VP16 overexpression efficiency (A and B, lower). Bar graph represents the result of DLRs from 3 independent experiments expressed as means ± SEM; (**c**, **d**) The promoter activity of *α0* and *α4* in the context of Hsp90α or Hsp90β downregulation. Vero cells were transfected with reporter plasmids together with siHsp90α or siHsp90β (100 nM) as indicated for as described in section 2.4 of the Materials and Methods. Cell lysates were then harvested to test luciferase activity. Bar graph represents the result of DLRs from 3 independent experiments expressed as means ± SEM; (**e**) Effects of Hsp90α or Hsp90β knockdown on the RNA levels of *α0* and *α4*. Vero cells were transfected with siHsp90α-2, siHsp90α-3, and siHsp90β (100 nM) for 24 h and then infected with HSV-1 (MOI 50). Total RNA was extracted at 2 hpi and then subjected to the analysis of RNA levels of *α0* and *α4* using qRT-PCR. **f** Hsp90α restored the suppressed promoter activity of *α0* and *α4* genes induced by Hsp90α knockdown. Vero cells were cotransfected with siHsp90α-2 (100 nM) and the indicated luciferase reporter plasmids as described in section 2.4 of the Materials and Methods. The cell lysates were subjected to luciferase activity assays. Bar graph represents the result of DLRs from three independent experiments expressed as means ± SEM; (**g**) Plaque formation assays showing that HSV-1 infection was affected by Hsp90α knockdown. Cells were infected with HSV-1 for 2 h after transfecting siHsp90α or siHsp90β (100 nM) knockdown and then subjected to plaque assays (left). Quantitative histograms were prepared (right). **h** Virus yield assay in the context of Hsp90 knockdown and Hsp90 inhibition. Cells were infected with HSV-1(MOI 1) for 24 h after transfecting siHsp90α (100 nM) and then subjected to determine the yield of virus; the group of acyclovir was also included as a control. **i** Hsp90 inhibition reduced the fluorescenceintensity of GFP-HSV-1 infected SH-SY5Y cells; SH-SY5Y cells were infected with GFP-HSV-1 (MOI 1) for 24 h in the presence of 17AAG (0.5 μM) or AT533 (2 μM). The cells were observed with fluorescence microscope. Scale bars, 200 μm

Our previous studies showed that Hsp90 inhibition greatly attenuates HSV-1 infection and the expression of HSV-1 α genes (Xiang et al., 2012a; Zhong et al., 2014). In the present study, we confirmed that the transcription of two other α genes, *α0* and *α4,* was also suppressed by Hsp90 inhibition (Additional file 1: Figure S1A and S1B). Dual luminal assays were subsequently performed in order to test whether Hsp90 was involved in modulating the promoter activity of *α0* and *α4*, which contain VP16 associated transactivator complex binding sites (Wysocka & Herr, 2003; Liang et al., 1999; Guo et al., 2012). Hsp90 inhibition suppressed luciferase activity in the pcDNA3.1(+)-VP16 plasmid-transfected group but did not affect luciferase activity in the control group that was transfected with an empty vector (Fig. 1a and b, upper). Corresponding VP16 protein levels were measured to assess the efficacy of VP16 expression from the plasmids (Fig. 1a and b, lower). To further determine the specific Hsp90 isoform required for modulating promoter activity of *α0* and *α4*, siRNAs that efficiently targeted Hsp90α or Hsp90β were used. Interestingly, knockdown of Hsp90α suppressed the promoter activity of α genes, whereas Hsp90β knockdown had no significant effect (Fig. 1c and d). The corresponding efficacy of siRNA knockdown was assessed by western-blot (Additional file 1: Figure S1C). Consistently, the RNA levels of both *α0* and *α4* were significantly reduced in different HSV-1-infected Hsp90α-knockdown cells, whereas there was no such effect in different Hsp90β-knockdown cells, including SH-SY5Y (Fig. 1e) and Vero (Additional file 1: Figure S1D) cells. Moreover, Hsp90α overexpression restored the Hsp90 downregulation-induced-suppression of the promoter activity of α genes, providing further evidence that Hsp90α was involved in the transactivation of HSV-1 α genes (Fig. 1f). However, Hsp90α overexpression alone failed to enhance the expression of α genes and promoter activity, indicating that Hsp90α was a coactivator rather than a direct activator of HSV-1 α genes (Fig. 1f and Additional file 1: Figure S1E). Consistent with these findings, both the plaque titers (Fig. 1g) and the yields of virus (Fig. 1h) were significantly reduced in HSV-1-infected cells transfected with siRNA against Hsp90α. To determine whether this effect was virus-strain specific, we tested the antiviral activity of Hsp90 inhibitors on GFP-HSV-1, another HSV-1 strain that harbors GFP-tagged U_S_11. The results indicated that the intensity of GFP fluorescence was significantly reduced in the presence of AT533 as compared to that in the control group (Fig. 1i).

### Hsp90α is required for the maintenance of VP16 stability

**Fig. 2:**
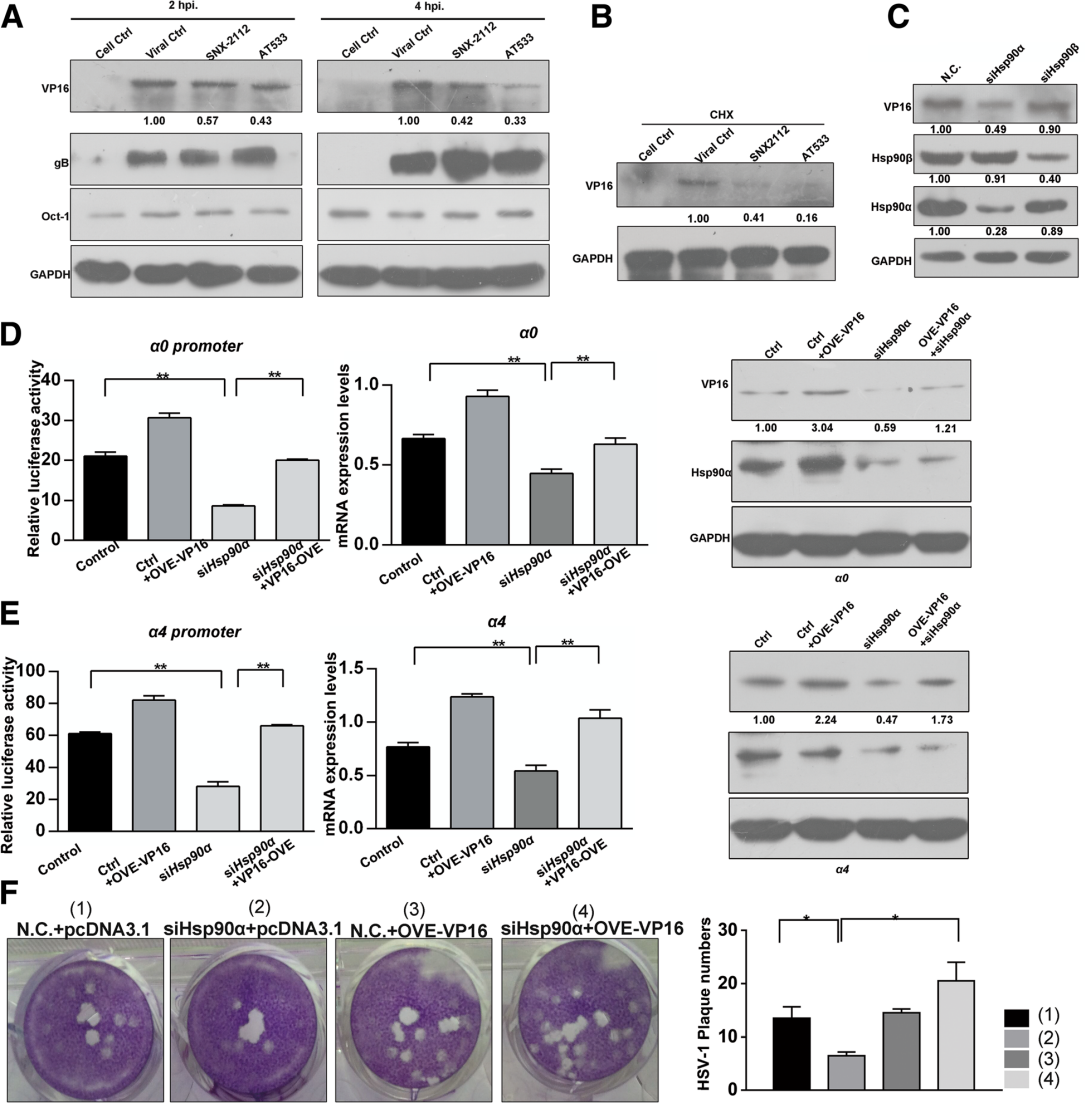
Hsp90α is required for the maintenance of the stability of VP16. **a** Hsp90 inhibition reduced VP16 protein levels in HSV-1-infected cells. Vero cells were infected with HSV-1 (MOI 50) for the indicated times in the presence of SNX-2112 (0.8 μM) or AT533 (2 μM), and total protein was extracted for western blot analysis to detect VP16 and gB. **b** Vero cells were infected with HSV-1 (MOI 50) in the presence of cycloheximide (100 μg/mL) and SNX-2112 (0.8 μM) or AT533 (2 μM), and protein was then extracted at 2 hpi for western blot analysis. **c** Hsp90α knockdown, but not Hsp90β knockdown, led to VP16 degradation. SH-SY5Y cells were transfected with siHsp90α-2 or siHsp90β (100 nM) for 24 h and then infected with HSV-1 (MOI 50) for 2 h. Protein samples were extracted and then subjected to western blot analysis. **d** VP16 restored the suppressed promoter activity of *α0* and *α4* genes induced by Hsp90α knockdown. Vero cells were cotransfected with siHsp90α-2 (100 nM) and the luciferase reporter plasmid as described in section 2.4 of the Materials and Methods. The cell lysates were subjected to luciferase activity assays (left panel) and analyzed by western blotting for detection of Hsp90α and VP16 (right panel). The results were provided as means ± SEM that calculated from three independent experiments. **e** Vero cells were cotransfected with siHsp90α-2 (100 nM) and pcDNA-VP16 plasmids (3 μg) for 48 h and then infected with HSV-1 (MOI 50) for 2 h. Total RNA was extracted for analysis of *α0* and *α4* RNA levels by qRT-PCR (left panel); Vero cells were cotransfected with siHsp90α-2 (100 nM) and pcDNA-VP16 plasmids (3 μg) for 48 h and then infected with HSV-1 (MOI 50) for 2 h. Total proteins were also extracted to detect VP16 and Hsp90α (right panel). **f** VP16 overexpression restored the reduction of HSV-1 infection mediated plaque formation that Hsp90α knockdown induced. Cells were infected with HSV-1 (MOI 0.1) for 2 h after co-transfection with siHsp90α-2 (100 nM) and pcDNA-VP16 plasmids (3 μg) and then subjected to plaque assays (left). Quantitative histograms were prepared (right)

Based on the fact that VP16 is a core factor in the transcription of HSV-1 α genes and the findings that VP16 expression from plasmids was reduced following treatment with different Hsp90 inhibitors, including 17AAG and AT533 (Fig. 1a), we speculated that VP16 may be a crucial mediator in the regulation of HSV-1 α genes transactivation by Hsp90. We first performed immunofluorescence assays to detect the level of VP16 in the presence of Hsp90 inhibitors. The results indicated that the inhibition of Hsp90 did not alter the subcellular localization of VP16 (Additional file 1: Figure S2) and that Hsp90 and VP16 partially colocalized in infected cells in the absence of Hsp90. Moreover, in the presence of different Hsp90 inhibitors, including AT533 and SNX-2112, VP16 was significantly reduced at the protein level in HSV-1-infected cells at 2 h and 4 h post infection (hpi; Fig. 2a). However, levels of glycoprotein B (gB), another component of HSV-1 viral particles, and levels of Oct-1, a crucial host transactivation factor, were not significantly altered, thereby largely reducing the possibility that Hsp90 inhibitors affected other components at this phase (Fig. 2a). We also detected VP16 mRNA expression at the indicated times and found no significant change in RNA levels although there was a slight trend of reduction (Additional file 1: Figure S3). This excluded an effect of HSP90 inhibition on VP16 transcription. To analyze the sole effect of input VP16, cycloheximide (CHX) was used to block protein translation and revealed that levels of input VP16 still significantly decreased in cells treated with Hsp90 inhibitors, including AT533 and SNX-2112 (Fig. 2b). Moreover, Hsp90α knockdown also led to the degradation of VP16 in HSV-1-infected cells, whereas Hsp90β knockdown caused no significant changes (Fig. 2c). Furthermore, VP16 overexpression abolished the Hsp90α knockdown-induced suppression of α gene promoter activity and restored the reduction in VP16 that was induced by Hsp90α knockdown (Fig. 2d). Consistent with this, the overexpression of VP16 in HSV-1-infected cells also restored the reduced mRNA levels of α genes and the loss of VP16 in the presence of AT533 (Fig. 2e). VP16 overexpression also restored reduction in Hsp90α knockdown-induced plaque formation **(**Fig. 2f). Overall, these findings indicated that Hsp90α was required for maintaining the stability of the VP16, which is associated with the transcription of HSV-1 α genes.

### Hsp90 inhibition induced autophagy-mediated degradation of VP16

**Fig. 3:**
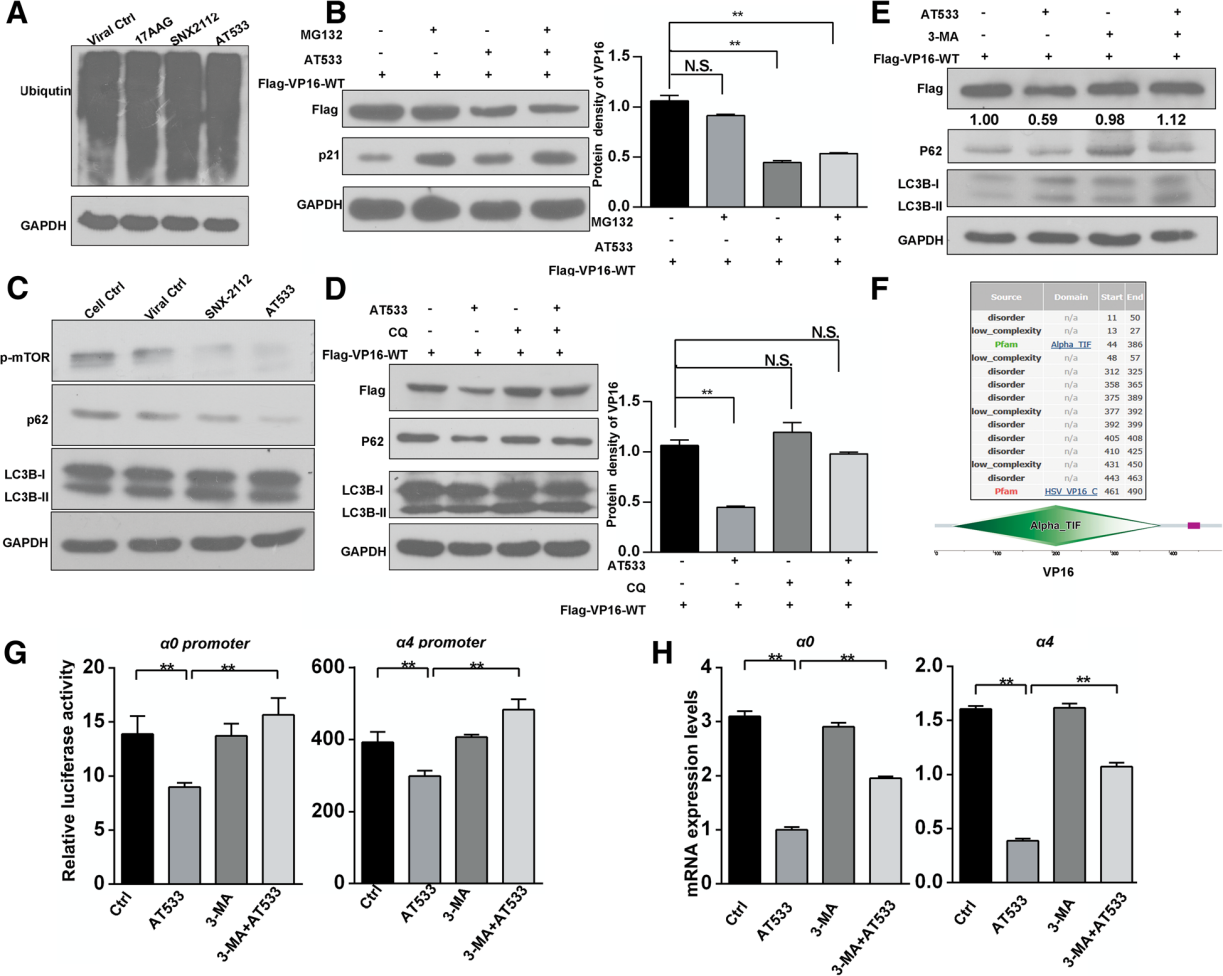
Hsp90 inhibition mediates autophagy-dependent degradation of VP16. **a** Hsp90 inhibitors induce the accumulation of endogenous ubiquitinated proteins. Vero cells were infected with HSV-1 (MOI 50) for 2 h in the presence of 17AAG (0.5 μM), SNX-2112 (0.8 μM), or AT533 (2 μM), and total protein was extracted for western blot analysis. **b** MG132 failed to rescue the loss of VP16 induced by Hsp90 inhibition. Vero cells were transfected with FLAG-VP16 plasmid (3 μg) for 48 h and treated with AT533 (2 μM) for 2 h in the presence of MG132 (5 μM). Total protein samples were extracted and subjected to western blot analysis (left). Notably, the accumulation of p21, a cellular protein degraded by the proteasome, was included as an indicator of proteasome activity inhibition. Quantify One densitometric analysis of VP16 band from FLAG-immunoblots (means ± SD of 3 independent experiments) (right). **c** Hsp90 inhibitors activated the macro-autophagy pathway in HSV-1-infected cells. Vero cells were infected with HSV-1 (MOI 50) for 2 h in the presence of SNX-2112 (0.8 μM) or AT533 (2 μM) and then analyzed by western blotting to determine the expression of mTOR pathway-associated proteins; P62 is an autophagy receptor that interacts directly with both the cargo to become degraded. **d** CQ rescued the loss of VP16 induced by Hsp90 inhibition. Vero cells were transfected with FLAG-VP16 plasmid (3 μg) for 48 h and treated with AT533 (2 μM) for 2 h in the presence of CQ (50 μM). Protein was extracted, and protein expression of VP16 was analyzed by western blotting (left). Quantify One densitometric analysis of VP16 band from FLAG-immunoblots (means ± SD of 3 independent experiments) (right). **e** 3-MA restored the loss of VP16 protein induced by Hsp90 inhibition. Vero cells were transfected with FLAG-VP16 plasmid (3 μg) for 48 h and then pretreated with 3-MA (5 mM) for 3 h before treatment with AT533 (2 μM) for 2 h, as indicated. The samples were then analyzed by western blotting. **f** The SMART diagram represents the major domains within VP16 that were obtained from UniProt (upper). Additional information regarding the predicted domains is also provided as a Table (lower). **g**, **h** 3-MA restored the suppressed promoter activity and downregulation of *α0* and *α4* genes in HSV-1-infected cells. **g**, **h** Vero cells were transfected with indicated reporter plasmids for 18 h, pretreated with 3-MA (5 mM) for 3 h, and then treated with AT533 (2 μM) for 2 h. Cell lysates were subjected to luciferase activity assays. Bar graph represents the result of DLRs from 3 independent experiments expressed as means ± SEM. **h** Vero cells were pretreated with 3-MA (5 mM) for 3 h and infected with HSV-1 (MOI 50) for 2 h in the presence of AT533 (2 μM). Total RNA was then extracted and subjected to qRT-PCR analysis

Next, we attempted to identify the specific mechanisms responsible for the Hsp90 inhibition-induced VP16 degradation. In our previous study and those of others, Hsp90 inhibition has been shown to promote the ubiquitination of Hsp90 client proteins (Liu et al., 2012; Gao & Harhaj, 2013; Chen et al., 2012). Thus, we first assessed the levels of ubiquitinated proteins in cells treated with different Hsp90 inhibitors and found that all treatments induced the accumulation of polyubiquitin proteins (Fig. 3a). Next, MG132, a specific proteasome inhibitor, was used to examine the role of the proteasome pathway in Hsp90 inhibition-induced degradation of VP16. In the presence of MG132, treatment with AT533 still led to VP16 degradation in VP16-expressing cells (Fig. 3b). The accumulation of p21 in the presence of MG132 was included as a positive indicator of suppressed protease activity, suggesting that the proteasome pathway was not involved in VP16 degradation. Moreover, no treatments significantly affected *VP16* mRNA levels, excluding the possibility that VP16 transcription was affected (Additional file 1: Figure S4A). In addition, since Hsp90 inhibitors have been suggested to activate the autophagy pathway (Liu et al., 2016; Liu et al., 2012; Mori et al., 2015; He et al., 2016), we examined whether Hsp90 inhibition-induced VP16 degradation was dependent on the autophagy pathway. Lysosomal turnover of LC3B-II from LC3B-I reflects autophagic activity as LC3B-II indicates the formation and lengthening of the autophagosome (Tanida et al., 2008). Consistent with previous reports, we found that treatment of cells with Hsp90 inhibitors, including SNX2112 and AT533, activated autophagy by reducing the level of mammalian target of rapamycin (mTOR), a classical signaling pathway that suppresses autophagy (Liu et al., 2012; Mori et al., 2015; He et al., 2016) (Fig. 3c). Confocal images of LC3B puncta also indicated that Hsp90 inhibitors enhanced autophagosome formation (Additional file 1: Figure S4B). Chloroquine (CQ), an inhibitor of the fusion of the lysosome and autophagosome (Beatman et al., 2012), reversed the degradation of VP16 mediated by Hsp90 inhibition (Fig. 3d) by maintaining a consistent level of *VP16* mRNA (Additional file 1: Figure S4C). This suggested that the Hsp90 inhibition-induced degradation of VP16 was dependent on the autolysosomal degradation pathway.

However, two main autophagic proteolysis pathways, the chaperone-mediated autophagy (CMA) pathway and the macro-autophagy (MA) pathway, can be activated by Hsp90 inhibitors (Finn et al., 2005; Dice, 2007). Autophagy also can be inhibited by 3-MA by inactivating phosphatidylinositol 3-kinase (PI3K), which is required for membrane trafficking during autophagy but has no effect on the activation of CMA (Finn et al., 2005; Verschooten et al., 2012; Petiot et al., 2000; Blommaart et al., 1997). Thus, 3-MA was used to block AT533-induced autophagy, which restored the AT533-induced loss of VP16 (Fig. 3e). Moreover, the UniProt database indicates that VP16 lacks the corresponding domains required by CMA, such as the loose pentapeptide motif KFERQ or biochemically related sequences (KFERQ-like) (Fig. 3f). Hence, VP16 degradation mediated by Hsp90 inhibition may be due to MA. Finally, to confirm that autophagy participated in Hsp90 inhibition-mediated suppression of α gene transcription, dual luminal rescue assays were performed in combination treatment with AT533 and 3-MA or CQ. Notably, 3-MA restored the suppression of α gene promoter activity and the downregulation of α genes in the presence of AT533 (Fig. 3g and h). Unexpectedly, CQ failed to rescue the Hsp90 inhibition-induced suppressive activity of α gene promoters and the transcriptional suppression of the *α0* and *α4* genes in HSV-1-infected cells (Additional file 1: Figure S5).

### VP16 interacted with Hsp90α via its conserved core domain

**Fig. 4:**
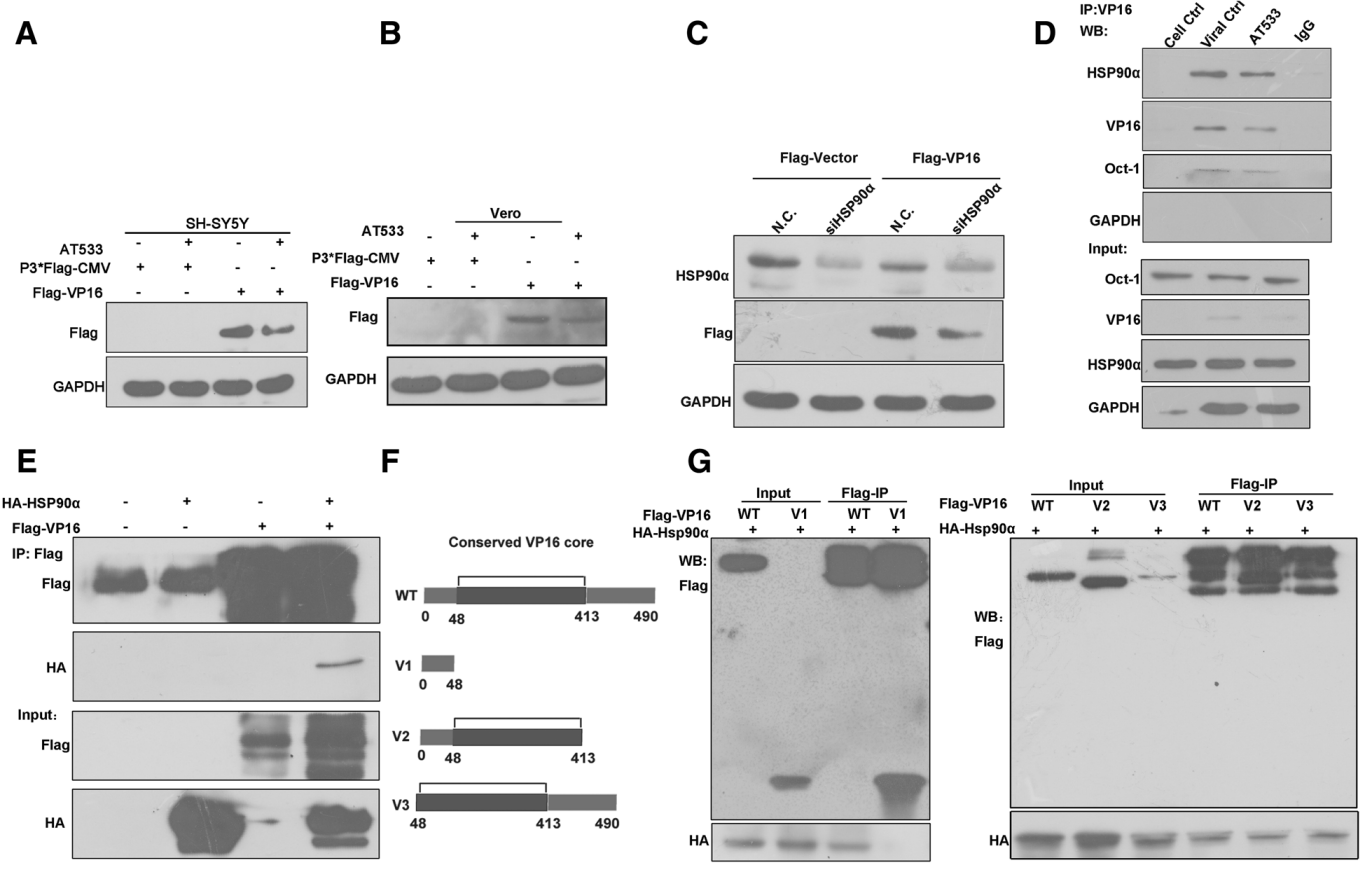
VP16 interacts with Hsp90α via its conserved core domain. **a**, **b** Hsp90 inhibition led to VP16 degradation in FLAG-VP16 plasmid-transfected cells. SH-SY5Y cells (**a**) and Vero cells (**b**) were transfected with FLAG-VP16 plasmid (3 μg) for 48 h and then treated with AT533 (2 μM) for 2 h. Proteins were extracted and subjected to western blot analysis; P3*FLAG-CMV was included as an empty vector control. **c** Hsp90α knockdown contributed to VP16 degradation in VP16-expressing cells. SH-SY5Y cells were cotransfected with siHsp90α-2 (100 nM) and FLAG-VP16 plasmid (3 μg) for 48 h. The cell lysates were then analyzed by western blotting. **d** SH-SY5Y cells were infected with HSV-1 (MOI 50) for 2 h in the presence of AT533 (2 μM), immunoprecipitated with anti-VP16 antibodies, and subjected to western blot analysis using anti-Hsp90 antibodies. Oct-1 was used as a positive indicator that interacts with VP16. **e** VP16 interacted with Hsp90α in 293 T cells. 293 T cells were cotransfected with FLAG-VP16 (5 μg) and HA-Hsp90α (5 μg) plasmids for 48 h. The cells were then lysed, subjected to immunoprecipitation using anti-FLAG antibodies, and then analyzed by western blotting. **f** Diagrammatic sketch of VP16 truncated mutant construction. **g** VP16 interacted with Hsp90α via its conserved core domain. 293 T cells were cotransfected with different truncated mutant plasmids of VP16 (5 μg), including WT, V1, V2, and V3, together with HA-Hsp90α (5 μg) plasmid for 48 h. The cells were lysed and subjected to immunoprecipitation using anti-FLAG antibodies and then analyzed by western blotting

The client proteins of Hsp90 would are degraded when Hsp90 activity is inhibited (Chen et al., 2012; Wang et al., 2016b; Abufarha et al., 2011). Thus, we attempted to determine whether VP16 was a client protein of Hsp90. We constructed the pFLAG-CMV-10-VP16 (FLAG-VP16) and pCMV-HA-Hsp90α (HA-Hsp90α) plasmids in order to determine the stability of VP16 with Hsp90 inhibition outside the context of HSV-1 infection. We found that VP16 was downregulated following Hsp90 inhibition in different FLAG-VP16 plasmid-transfected cell lines (Fig. 4a and b). Consistently, the amount of VP16 expressed from transfected plasmids was also significantly reduced in Hsp90α-knockdown cells (Fig. 4c).

We also performed endogenous immunoprecipitation in HSV-1-infected SH-SY5Y cells and found that VP16 interacted with Hsp90α in HSV-1-infected cells, and that the level of VP16 was reduced by treatment with the Hsp90 inhibitor AT533 (Fig. 4d). Moreover, exogenous immunoprecipitation assays were performed in 293 T cells in which we found a significant positive interaction between Hsp90α and VP16 (Fig. 4e). To identify the interacting domain between Hsp90α and VP16, truncated mutants of VP16 were constructed as shown in Fig. 4f (Liang et al., 1999). We co-transfected plasmids carrying different FLAG-tagged truncated mutants of VP16 with HA-Hsp90α plasmids and then performed anti-FLAG immunoprecipitation. The truncated mutants lacking the 48 amino acids of the N-terminus (V2) and/or the 78 amino acids of the C-terminus (V3) still interacted with Hsp90α, whereas the truncated mutant V1, which lacked the conserved core domain, failed to interact with Hsp90α (Fig. 4g). Taken together, these results indicated that VP16 was a client protein of Hsp90α and interacted with Hsp90α via its conserved core domain.

### Hsp90 inhibition induced VP16 degradation is dependent on the conserved core domain of VP16

**Fig. 5:**
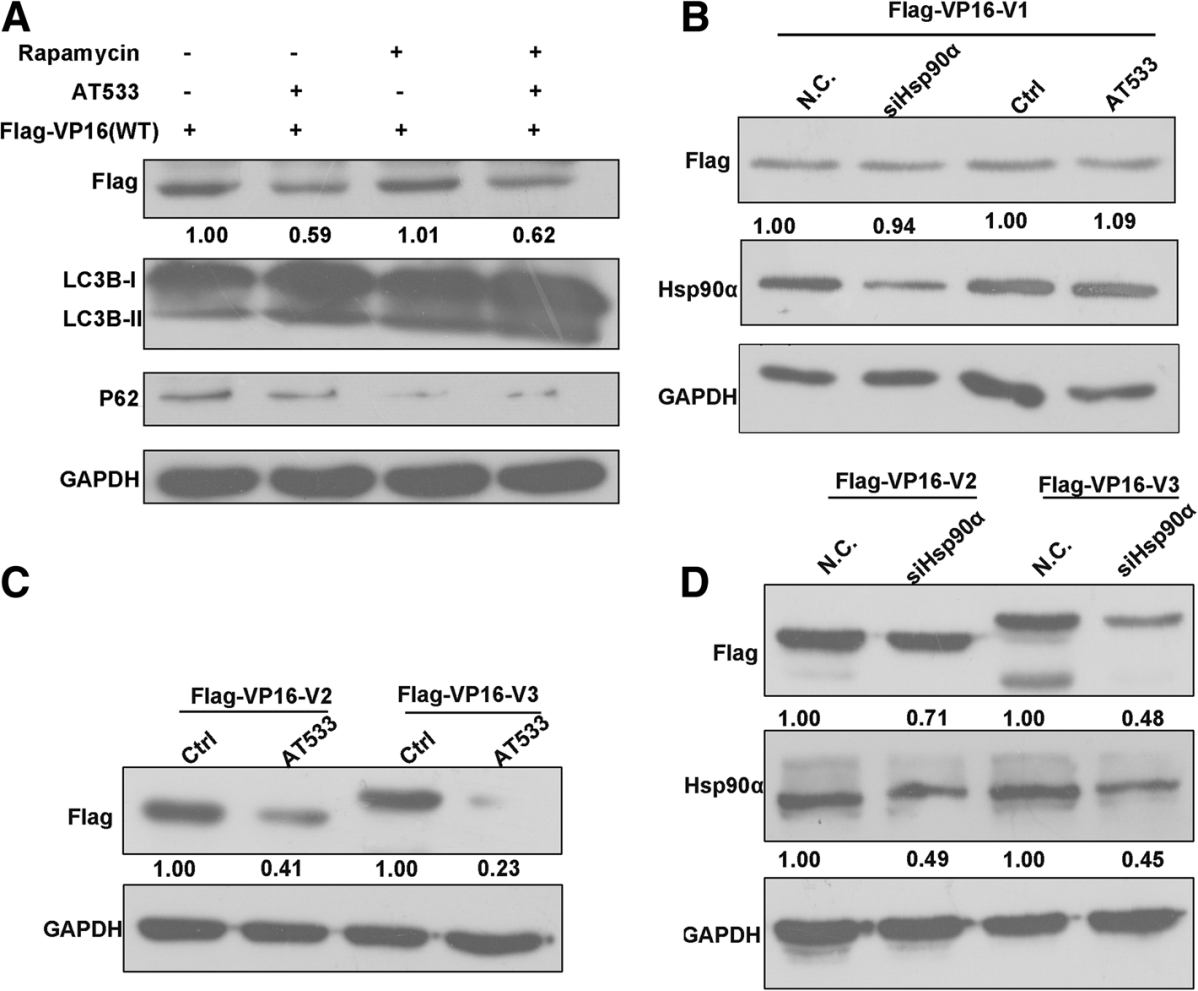
Hsp90 inhibition-induced VP16 degradation depends on the conserved core domain of VP16. **a** Treatment with rapamycin failed to lead to VP16 degradation. Vero cells were transfected with FLAG-VP16 plasmid (3 μg) for 36 h, treated with rapamycin (250 nM) for 10 h, and then treated with AT533 (2 μM) for another 2 h. Total proteins were extracted and analyzed by western blotting. **b** Hsp90α knockdown and inhibition did not affect the level of mutant V1. Vero cells were cotransfected with siHsp90α-2 (100 nM) and the mutant FLAG-VP16 plasmid V1 (3 μg) for 48 h, and total proteins were extracted for western blot analysis (left). Vero cells were transfected with the mutant FLAG-VP16 plasmid V1 (3 μg) for 48 h and then treated with AT533 (2 μM) for 2 h. Total proteins were extracted and analyzed by western blotting (right). **c** Knockdown of Hsp90α reduced the levels of truncated mutants V2 and V3. SiHsp90α-2 (100 nM) was cotransfected with the mutant plasmid V2 or V3 (3 μg) into Vero cells for 48 h, and the levels of the indicated proteins were analyzed by western blotting. **d** Vero cells were transfected with the truncated mutant plasmid V2 or V3 (3 μg) for 46 h and then treated with AT533 (2 μM) for another 2 h. Total protein was extracted and the expression levels of the proteins of interest were analyzed by western blotting

Given that the sole treatment of cells with Hsp90 inhibitors is sufficient to activate autophagy pathway, we next attempted to determine whether VP16 degradation was a result of activated autophagy alone or due to the disassociation of the interaction between VP16 and Hsp90 that resulted from treatment with Hsp90 inhibitors (Liu et al., 2012; Mori et al., 2015; He et al., 2016). Rapamycin, which positively regulates autophagy by inhibiting mTOR (Blommaart et al., 1995), was utilized to activate autophagy in VP16-expressing cells. We found that treatment with rapamycin alone failed to lead to VP16 degradation; thus, the activation of autophagy alone was unable to degrade VP16 (Fig. 5a). In addition, combining the treatments of AT533 and rapamycin failed to enhance the degradation of VP16(Fig. 5a), suggesting that the targets of AT533 and rapamycin may function similarly. Subsequently, we transfected the different truncated mutant plasmids of VP16 into cells and compared their stabilities in the presence of AT533 or in the context of Hsp90α knockdown. No significant reductions in the levels of the truncated mutant V1 were found in the presence of AT533, although AT533 still contributed to the degradation of truncated mutants that interacted with Hsp90α, including V2 and V3 (Fig. 5b and c). Similar results were also found in Hsp90α-knockdown cells using Hsp90α siRNA (Fig. 5b and d). Taken together, Hsp90 inhibition-induced VP16 degradation was dependent on the conserved core domain of VP16.

### Hsp90 inhibition ameliorated HSV-1 infection-induced skin lesions in mice

**Fig. 6:**
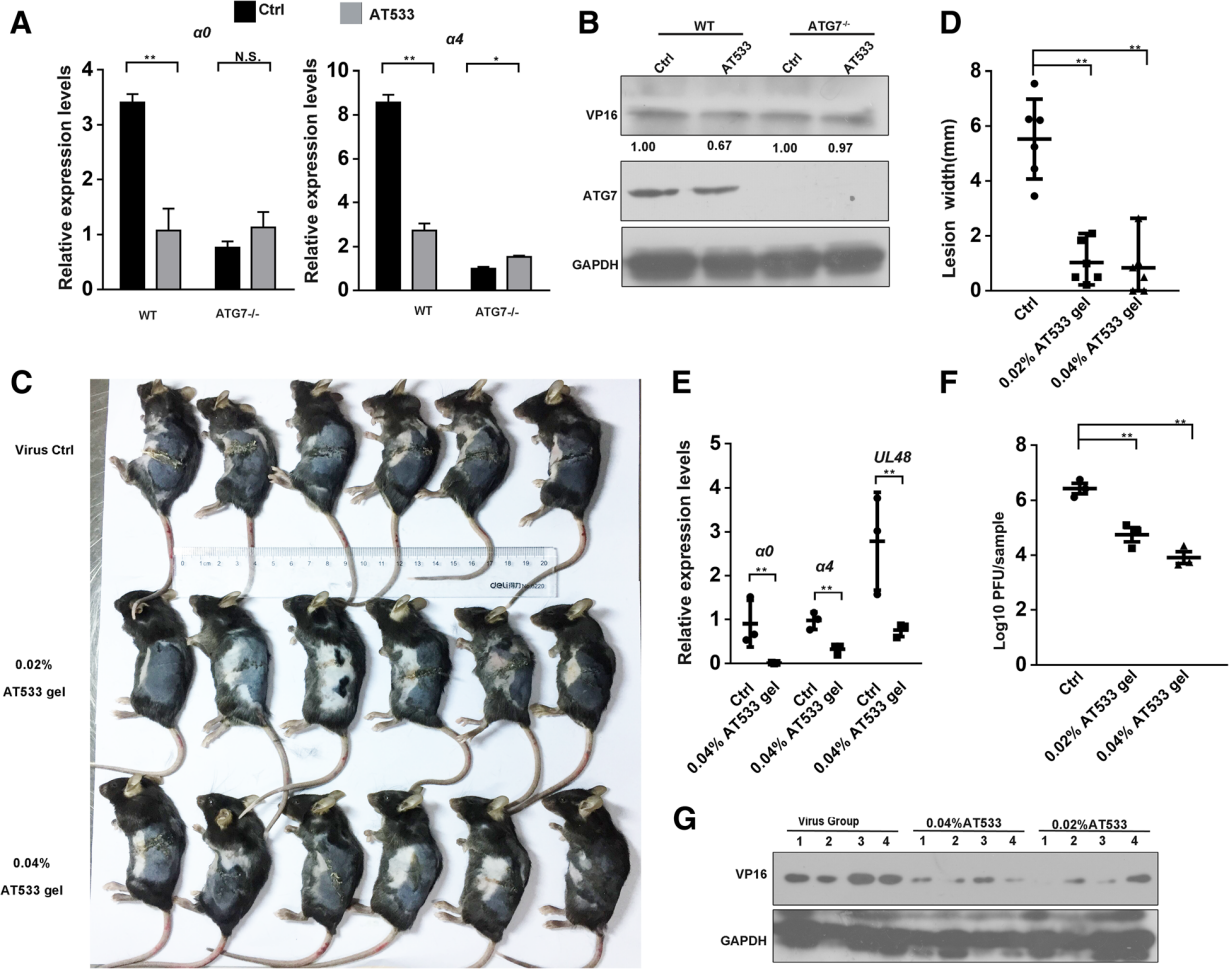
Hsp90 inhibition ameliorated HSV-1 infection-induced skin lesions in mice. **a**, **b** Hsp90 inhibitors failed to reduce α gene expression and led to VP16 degradation in autophagy-deficient cells. **a** MEF WT and ATG7^−/−^ cells were infected with HSV-1 (MOI 50) for 2 h in the presence or absence of AT533 (2 μM) or not and then total RNA was extracted and analyze by qRT-PCR to determine the level of α gene expression, including *α0* and *α4*. **b** MEF WT and ATG7^−/−^ cells were infected with HSV-1 (MOI 50) for 2 h in the presence of AT533 (2 μM), and total protein was extracted for western blot analysis to detect the level of VP16. **c** HSV-1-infected mice were treated with gels containing the indicated concentrations of AT533 on the infected skin once per day. Mice were sacrificed on day 7 after HSV-1 infection and then the zosteriform lesions were evaluated and snapped (**c**). The size of the zosteriform lesions was quantified by determining the widths of the zosteriform lesions from three mice and are expressed as means ± SD (D). **e** Approximately a 1 cm square piece of skin tissue was obtained as described in section 2.9 of the Materials and Methods, and total RNA was extracted. The relative expression of the *α0*, *α4* and VP16 genes was determined by real-time PCR (*n* = 3 mice). **f** Approximately a 1 cm square piece of skin tissue was obtained and then analyzed for viral titers with the method described in section 2.5 of the Materials and Methods (*n* = 3 mice). **g** The corresponding skin samples were obtained from mice as described in section 2.9 of the Materials and Methods then the total protein was extracted to analyze the level of VP16 using western blotting (*n* = 4 mice)

Before performing in vivo assays in mice, we tested the effect of an Hsp90 inhibitor on the expression of HSV-1 α genes and VP16 level in HSV-1-infected mouse cell lines, including wild-type (WT) and autophagy-deficient ATG7^−/−^ mouse embryonic fibroblast (MEF) cells. The results showed that Hsp90 inhibitors reduced the expression of α genes in HSV-1-infected WT MEF cells, similar to effects observed in SH-SY5Y and Vero cells (Fig. 6a). Distinct from the effect observed in WT MEF cells, no significant reduction was found in VP16 or α gene levels in HSV-1-infected ATG7^−/−^ MEF cells treated with Hsp90 inhibitors, suggesting that autophagy was required for Hsp90 inhibition-induced VP16 degradation (Fig. 6b). Given that our previous study confirmed that gels containing 0.025% AT533 displayed strong antiviral activity in a herpes stromal keratitis (HSK) rabbit model, we attempted to determine the efficacy of Hsp90 inhibitors in a zosteriform model of HSV-1 infection, another classic HSV-1 infection animal model (Xiang et al., 2012a; Van et al., 2004; Wang et al., 2016a). Gel mixture containing 0.02 and 0.04% AT533, were used to treat abraded skin once per day starting 24 h after HSV-1 infection once the skin lesions had significantly manifested. We found that compared to control mice treated with gels lacking drug, the gels containing AT533 significantly ameliorated the skin lesions especially in the group that received 0.04% AT533 (Fig. 6c). The relative size of the zosteriform lesions (Fig. 6d) and the virus titers in infected skin tissues (Fig. 6e) further supported the efficacy of the gels containing AT533 against HSV-1 infection. Furthermore, treatment with these gels profoundly reduced the level of HSV-1 α genes expression in HSV-1-infected skin tissues (Fig. 6f). In addition, western blot analysis indicated that VP16 was also significantly reduced by the treatment with Hsp90 inhibitors (Fig. 6g). These results indicated that Hsp90 inhibitor gels reduced the level of VP16 and α genes expression, which may contribute to suppressing the progression of zosteriform disease in vivo.

## Discussion

The function of the HSV-1 tegument protein VP16 is an absolutely essential requirement for a replication competent virus. Thus, the impact of Hsp90α on the VP16-mediated transactivation of α gene would be meaningful for the development of anti-HSV-1 drugs. Specifically, HSV-1 with mutant VP16 molecules, such as *in 1814*(the name of mutant HSV-1 strain) with an incapacitated VP16-induced complex formation or *RP54*(the name of mutant HSV-1 strain) with incapacitated for transcriptional activation, are almost avirulent in mice. In contrast, during infection of cultured cells with these viruses, viral infection is debilitated significantly but not completely abolished. Collectively, there may be two mechanisms for α genes transcription in HSV-1- infected cells, a VP16-dependent and a VP16-independent mechanism (Ace et al., 1989; Tal-Singer et al., 1999). Of note, such experiments performed in rodent cells underestimates the importance of VP16 because VP16-induced complex formation is weakened owing to a rodent-specific variation in Oct-1 (Wysocka & Herr, 2003). In our study, VP16 was incompletely degraded following Hsp90 inhibition, and Hsp90 inhibition may have no significant effect on the transactivation of VP16-independent α gene initiation. It is therefore reasonable to postulate that the reduction in Hsp90 protein levels in infected cells led to the 0.5-fold decrease in the expression of *α0* and *α4* in our study. Besides, VP16 is a multifunctional protein that can downregulate the virion shut-off protein and the production of IFN-β in RLR signaling and participate in egress downstream of the primary envelopment (Smibert et al., 1994; Lam et al., 1996; Mossman et al., 2000; Xing et al., 2013; Zheng & Su, 2017).

In the current study, we identified for the first time HSV-1 VP16 as a new client protein of Hsp90α. Immunoprecipitation assays indicated that VP16 interacted with Hsp90α and this interaction required the conserved core domain of VP16. Overexpression of VP16 rescued the Hsp90α inhibition- or knockdown-mediated reductions in *α0* and *α4* gene promoter activity and α gene transcription, suggesting that Hsp90α was involved in maintaining the stability of VP16 and the VP16-mediated transactivation of HSV-1 α genes. Notably, VP16 significantly enhanced the promoter activities of α genes, consistent with the observation that VP16 acts as a crucial factor in α gene transcription initiation (Wysocka & Herr, 2003; Liang et al., 1999; Guo et al., 2012; Triezenberg et al., 1988).

In addition, we identified autophagy as the major pathway of VP16 degradation that resulted from the inhibition of Hsp90. There are two protein degradation pathways, autophagy and the ubiquitin-proteasome pathway (Wong & Cuervo, 2010; Theodoraki & Caplan, 2012; Ciechanover, 2005). Hsp90 inhibition has been suggested to inactivate the mTOR pathway, a classical signaling pathway that suppresses autophagy (Liu et al., 2012; Mori et al., 2015; He et al., 2016), which was also confirmed in our study. Treatment with autophagy inhibitors, but not a proteasome inhibitor, reversed the degradation of VP16. However, both autophagy and CMA were activated by Hsp90 inhibitors; thus, further studies are performed to elucidate the specific autophagy pathways. The autophagy inhibitor, 3-MA, which does not influence CMA (Dice, 2007; Massey et al., 2006), reversed the loss of VP16 mediated by Hsp90 inhibition. During CMA, targeting proteins are recognized by HSPA8/HSC70, which depends on the pentapeptide motif KFERQ or biochemically related sequences (KFERQ-like). The substrate-chaperone complex is then translocated to the lysosomal membrane, where it interacts with LAMP-2A, a CMA-specific protein (Dice, 2007; Massey et al., 2006). However, structural analysis indicated that VP16 lacked the corresponding domain CMA required although the most effective method for measuring CMA activity is by testing the translocation of CMA substrates, such as LAMP-2A. Unlike in treatment with 3-MA, CQ treatment restored VP16 protein levels in the presence of Hsp90 inhibitors but failed to restore the reduced mRNA levels of the *α0* and *α4*. This may have been due to CQ alone reducing promoter activity and mRNA expression. There are no significant difference of the promoter activity and RNA level of *α0* and *α4* genes between the CQ treated group and the combination of CQ and AT533 treated group. Additionally, VP16 being wrapped into the autophagosome since CQ blocks the fusion of the lysosome with the autophagosome while 3-MA is an earlier autophagy inhibitor (Beatman et al., 2012). In contrast, in the presence of CQ, VP16 was probably still sequestered inside the autophagosomes but was.

not degraded. Therefore, VP16 was still detectable by Western blot while it is unavailable to form the VP16-induced complex and initiate IE gene expression.

Notably, no significant reduction in the levels of truncated mutant V1 was found in the context of Hsp90 inhibition and Hsp90α knockdown, while such treatment still led to the degradation of the truncated mutants that interacted with Hsp90α via the conserved domain within VP16, including V2 and V3. Moreover, the activation of autophagy with rapamycin alone was unable to degrade VP16. Therefore, Hsp90 inhibition may have led to VP16 degradation as a result of two aspects, the activation of autophagy and disruption of the interaction between VP16 and Hsp90α.

Furthermore, in our previous study, gels containing AT533 showed excellent efficacy against HSV-1 infection in an HSK rabbit model and were even more effective than ACV (Xiang et al., 2012a). Our present study demonstrated that Hsp90 inhibition with gels containing 0.02% or 0.04% AT533 remarkably improved skin zosteriforms caused by HSV-1 infection, suggesting that Hsp90 may be a promising therapeutic target for the treatment of HSV-1 infection. It is noteworthy that the anti-virus activity of Hsp90 inhibitors in vivo was more significant than that exhibited by the knockdown of Hsp90α. However, the efficacy of Hsp90 inhibitors and Hsp90 siRNA against Hsp90 may be different and the amount of virus used in vitro was far less than that used in vivo. Considering these results together, a two-fold reduction in plaque number mediated by the knockdown of Hsp90α may underestimate the efficacy of Hsp90 inhibitors against HSV-1 infection in vivo. Indeed, evaluation of mice on day 7 included the effect of the Hsp90 inhibitor on the viral DNA polymerase, suggesting that the effect of this experiment was not solely due to IE gene inhibition. Therefore, our in vivo assay solely provided additional mechanism of Hsp90 inhibition on HSV-1 infection, but not the only mechanism. Nevertheless, the in vivo data suggested that AT533 containing gels exhibited antiviral activity. Moreover, given that Hsp90 is involved in the regulation of HSV-1 α genes and that α gene production participates in the modulation of HSV-1 β and γ genes, it is reasonable to speculate that the mRNA level of *VP16* is also reduced following Hsp90 inhibition in HSV-1-infected skin tissues. Despite these findings, we failed to identify the specific role of Hsp90α in the formation process of transactivator complex. Therefore, even though we confirmed the interaction between Hsp90α and VP16, further studies are needed to determine whether Hsp90α facilitates the transportation of VP16 from the cytosol to the nucleus. It is also still unclear whether the interaction between Hsp90α and VP16 requires other factors. Moreover, we did not compare the levels of α genes with VP16-knockout and WT viruses treated with Hsp90 inhibitors, which would provide the most direct evidence in support of the impact of Hsp90 on the transactivation role of VP16. Nevertheless, as mentioned above, multiple studies have demonstrated that VP16 plays a crucial role in the HSV-1 life cycle; thus, analysis of the interaction between VP16 and Hsp90α would be meaningful for the further development of antiviral drugs.

## Conclusion

**Fig. 7:**
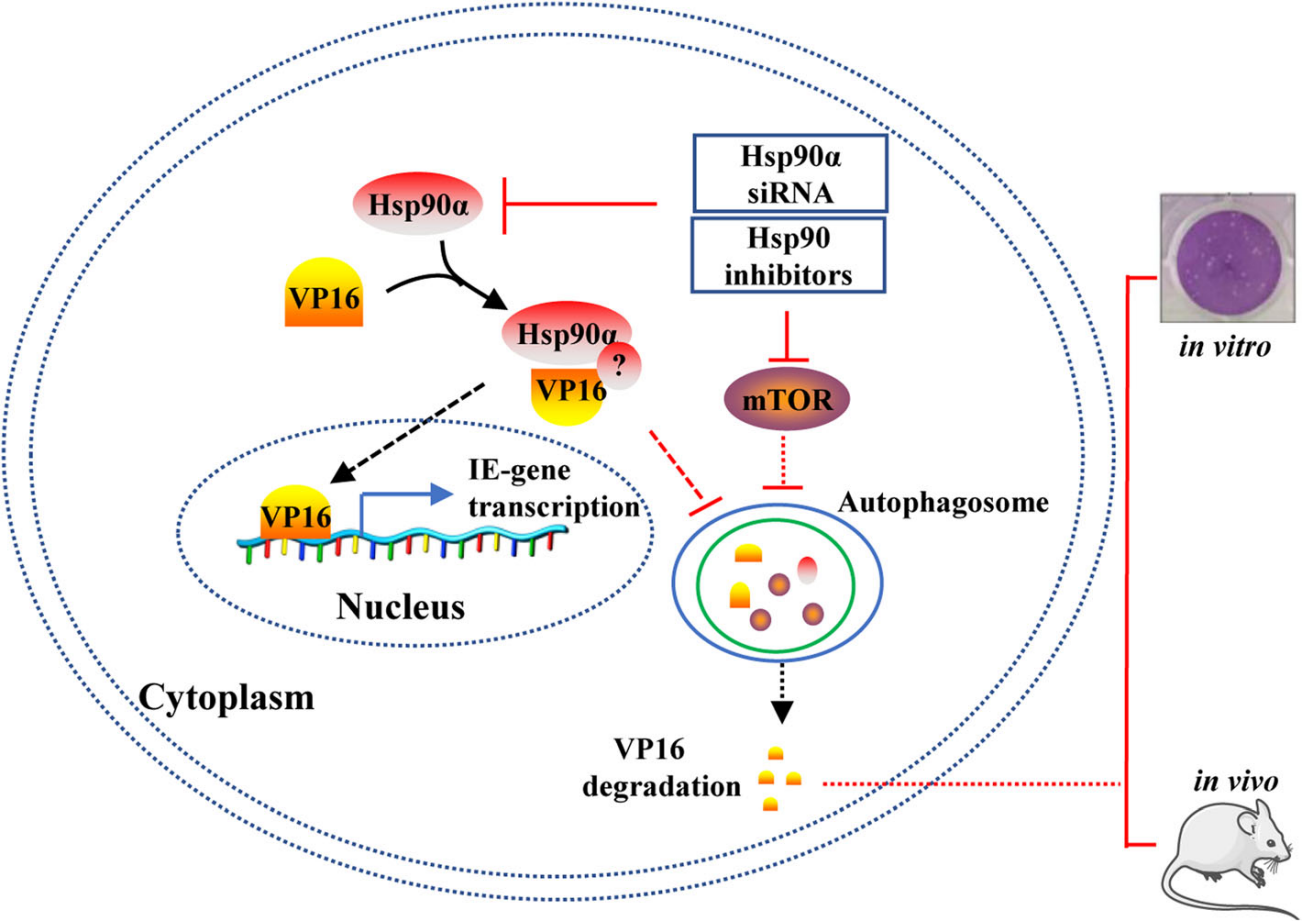
Schematic model of Hsp90α regulation of HSV-1 α genes in the context of Hsp90 inhibition or Hsp90α knockdown. Hsp90 inhibition or Hsp90α siRNA induces VP16 autophagosome degradation, leading to reduced α gene transcription, and then suppressed HSV-1 replication in vitro and in vivo. Specifically, Hsp90 inhibition disrupts the interaction between VP16 and Hsp90α and then induces the degradation in an autophagy-dependent manner. Notably, Hsp90 inhibition inhibits the mTOR pathway and then activates autophagy

Hsp90 inhibition has been reported to inhibit HSV-1 infection in our and other previous studies, but the major Hsp90 isoform that functions in the HSV-1 life cycle and the role of Hsp90 in the expression of the HSV-1 α genes remains unclear. Here, we found that Hsp90α, but not Hsp90β, was the major isoform used by HSV-1 to maintain the promoter activity of α genes and to ensure the VP16-mediated active transcription of *α0* and *α4*. A corresponding schematic diagram of the Hsp90α regulation model in HSV-1 α gene transcription is shown in Fig. 7. In brief, treatment of HSV-1-infected cells with Hsp90 inhibitors or Hsp90α knockdown by specific siRNA leads to activation of the autophagy pathway and disruption of the interaction between Hsp90 and VP16, which reduces the transcription of α genes associated with VP16 degradation. Our findings indicated that disruption of the interaction between Hsp90α and VP16 efficiently limited viral infection and may represent a promising strategy for reducing the toxicity of current Hsp90 inhibitors in the development of antiviral drugs.

## Statistical analyses

All qPCR results were obtained from three independent experiments and are expressed as means ± SEM. All statistical analyses were performed with Student’s two-tailed t-tests, with the level of significance set at *, *p* < .05; **, *p* < .01.

## Additional file

Additional file 1: Supplemental information includes three tables and five figures. (DOCX 765 kb)
