# Supplementary material for: Heat-shock protein 90α is involved in maintaining the stability of VP16 and VP16-mediated transactivation of α genes from herpes simplex virus-1
Source: Mol Med. 2018 Dec 22;24:65. doi: 10.1186/s10020-018-0066-x (PMC6303900; doi:10.1186/s10020-018-0066-x)
Supplement: Supplementary file 1 — Supplemental information includes three tables and five figures. (DOCX 765 kb) [file 10020_2018_66_MOESM1_ESM.docx]

**Supplemental Information**

Heat-shock protein 90α is involved in maintaining the stability of VP16 and VP16-mediated transactivation of α genes from herpes simplex virus-1

Yiliang Wang^a,b,c^, Rongze Wang^a, b,c,d^, Feng Li^a,b,c^, Yun Wang^e^, Zhen Zhang^a,b,c^, Qiaoli Wang^a,b,c^, Zhe Ren^a,b,c^, Fujun Jin ^a,b,c,g, #^, Kaio Kitazato^f, #^, and Yifei Wang^a,b,c, #^

**1. Supplemental methods**

**1. Laser scanning confocal immunofluorescence microscopy**

Cells were infected with HSV-1 for 2 h in the presence of Hsp90 inhibitors, then fixed in 4% paraformaldehyde-PBS for 15 min, permeabilized with 0.1% Triton X-100-PBS (Sigma-Aldrich) for 4 min, blocked with 5% bovine serum albumin-PBS for 1 h, and immunostained with primary mouse antibodies targeting HSV-1 VP16 (1:500) overnight. Cells were then stained with another primary rabbit antibody against Hsp90 (Abcam) for 2 h at room temperature. Subsequently, samples were probed with Alexa Fluor 488-conjugated anti-mouse antibodies (1:1000; Invitrogen) and Alexa Fluor 594-conjugated anti-rabbit antibodies (1:1000; Invitrogen) for another 1 h. Additionally, 1 mg/mL DAPI (BIOTIUM) was added to label nuclei for 15 min. Fluorescent images were captured with a Zeiss LSM510 Meta confocal system under a 63× oil immersion objective (Carl Zeiss). The fluorescence intensity of acquired images was analyzed and quantified using Zen software (Zeiss). Similarly, for confocal detection in FLAG-VP16 and HA-Hsp90α plasmid-transfected cells, the protocol was performed as previously described except that the antibodies targeted Flag and HA, respectively.

**2. Supplemental tables**

**Table S1. List of primers for plasmids construction.**

| **Plasmids** | **Restriction enzyme sites** | **Forward (5′ → 3′)** | **Reverse (5′ → 3′)** |
| --- | --- | --- | --- |
| pcDNA-VP16 | EcoRI + XhoI | CGGAATTCGGATGGACCTCTTG | CCGCTCGAGCTACCCACCGTACTC |
| P3*Flag*CMV10-VP16 | BamHI +EcoRI | GGAATTCAATGGACCTCTTGGTCGACGA | CGGGATCCCTACCCACCGTAC |
| pcDNA-human-HSP90α | BamHI+XbalI | CGGGATCCATTATGGCTGAGGAAACCCAGACCC | GCTCTAGATTAGTCTACTTCTTCCATGCGTGATGTGTCG |
| HA-human-HSP90α | SalI +KpnI | GCGTCGACCATGCCTGAGGAAAC | GGGGTACCTTAGTCTACTTCTTCCATGC |
| pGL-α0 promoter | KpnI+ XholI | GGGGTACCGTTCCGGGTTATGCTAATTGCTTTTTTG | CCGCTCGAGTGGCGCCCCAGACAT |
| pGL-α4promoter | KpnI+ XholI | GGGGTACCGGGCCCCGCCCCC | CCGCTCGAGGGATCCGTGTCGGCAGC |

**Table S2. Sequence of siRNAs that used in our study.**

| **Name** | **Sense** | **Antisense** |
| --- | --- | --- |
| **siHSP90α-2**  **(Monkey)** | GGAGCUAAUCCCUGAAUAU | AUAUUCAGGGAUUAGCUCC |
| **siHSP90α-3**  **(Monkey)** | UACUGCUCAUCAUCGUUAUGU | AUAACGAUGAUGAGCAGUACG |
| **siHSP90β** | CAGAAGACAAGGAGAAUUA | UAAUUCUCCUUGUCUUCUG |
| **siHSP90α-1(Human)** | TGGCGGACATTAAGGACATTG | TGGCCGTCAACTCGCAGA |
| **siHSP90α-2(Human)** | AGTCTGGGACCAAAGCGTTC | ACTGTGAATGATCCCCCTGC |

**Table S3. List of primers for qRT-PCR experiments.**

| **Gene** | **Primers (forward)** | **Primers (reverse)** |
| --- | --- | --- |
| *VP16* | TCGGCGTGGAAGAAACGAGAGA | CGAACGCACCCAAATCGACA |
| *α0* | CCCACTATCAGGTACACCAGCTT | CTGCGCTGCGACACCTT |
| *α4* | CGACACGGATCCACGACCC | GATCCCCCTCCCGCGCTTCGTCCG |
| *ICP27* | TGGCGGACATTAAGGACATTG | TGGCCGTCAACTCGCAGA |
| *HSP90α（Human）* | AGTCTGGGACCAAAGCGTTC | ACTGTGAATGATCCCCCTGC |
| *HSP90α（Monkey）* | CAGATGCATTGGACAAAATCCG | ATGAACGCTTTGGTCCCAGAC |
| *18s* | CATGGTGACCACGGGTGAC | TTCCTTGGATGTGGTAGCCG |
| *GAPDH(human)* | CACCATCTTCCAGGAGCGAG | AGAGGGGGCAGAGATGATGA |
| *GAPDH(mouse)* | TGTGTCCGTCGTGGATCTGA | CCTGCTTCACCACCTTCTTGA |

**3. Supplementary figure legends.**


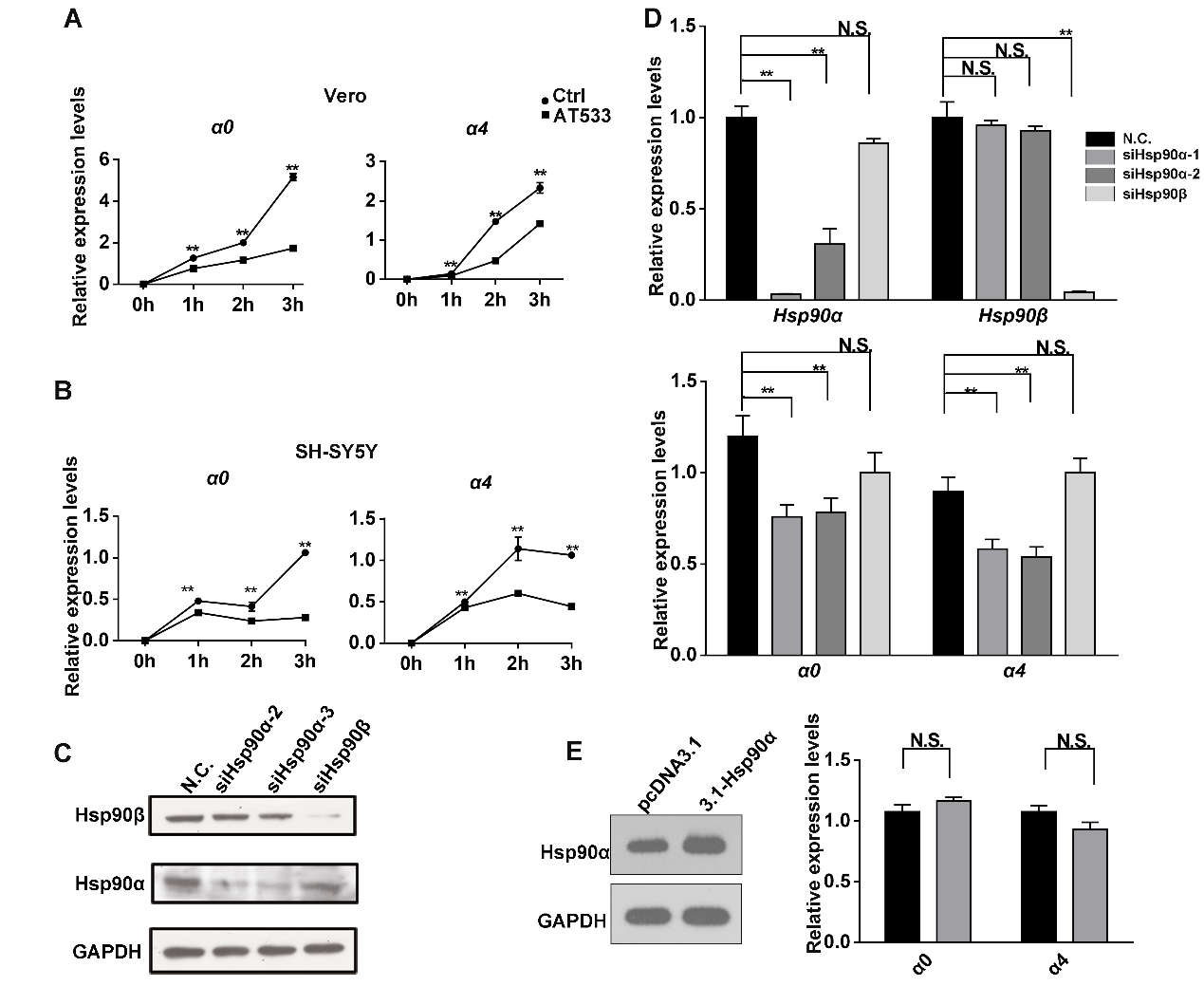


**Figure S1** SH-SY5Y**(A)** or Vero**(B)** cells were infected with HSV-1 (MOI 50) in the absence or presence of AT533 (2 μM) for indicated times, then total RNA was extracted to analysis of RNA levels of *α0* and *α4* using qRT-PCR**; (C)**Total proteins of the samples that were descripted in **Figure 1E** were extracted and then subjected to western blot analysis to assess the knockdown efficiency of siRNAs targeting Hsp90α and Hsp90β;**(D)**The RNA levels of *α0* and *α4* in HSV-1-infected SH-SY5Y cells in the context of Hsp90α or Hsp90β downregulation. All treatments were similar with the description in **Figure 1G** except for the cell types. **(E)** SH-SY5Y cells were transfected with the HA-Hsp90*α* plasmid (3 μg) for 48 h and then infected with HSV-1 (MOI 50) for 2 h. Total RNA was extracted to detect the RNA levels of *α0* and *α4* by qRT-PCR**(Right)**; The efficiency of Hsp90α overexpression was determined by western blot**(Left)**.


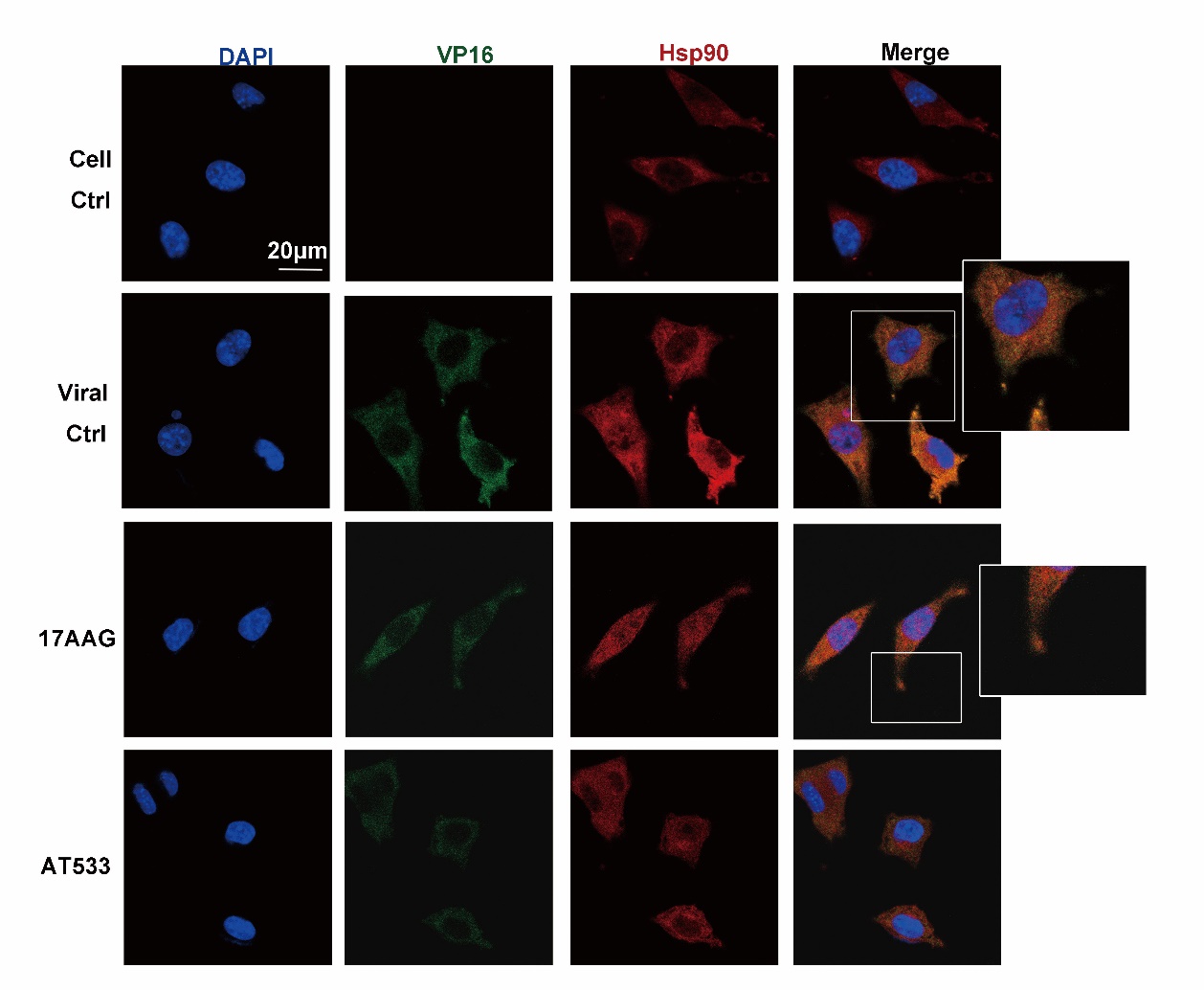


**Figure S2** Immunofluorescence staining of Hsp90 and VP16 in the presence of AT533 and 17AAG, respectively. Infected with HSV-1(MOI 50) in SH-SY5Y cells for 2 h then analyzed with immunofluorescence assay to detect Hsp90 and VP16.


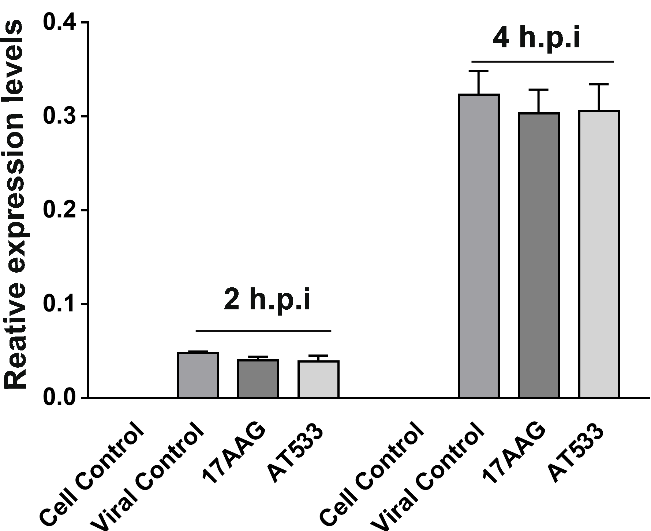


**Figure S3** Vero cells were infected with HSV-1 (MOI 50) for indicated times in the presence of 17AAG (0.8 μM) or AT533 (2 μM), and total RNA was extracted and then subjected to qRT-PCR analysis to detect the RNA level of VP16.


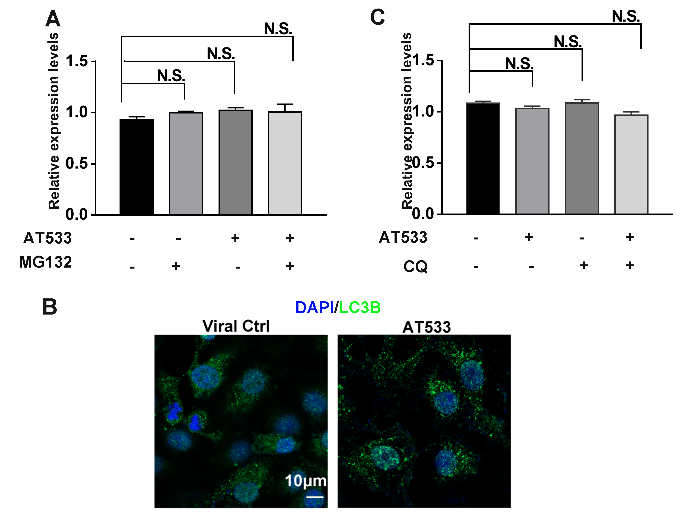


**Figure S4 A.** Total RNA of the samples in **Figure 4B** was extracted and then subjected to qRT-PCR for detection of VP16 RNA levels. **B**. SH-SY5Y cells were infected with HSV-1(MOI 50) for 2 h then fixed and labeled LC3B(*green*) and ICP5(*red*) with indicated antibody. The samples were processed for confocal microscopy image. ICP5 was used to ensure the cell was infected by HSV-1. **C.** Total RNA of the samples that described in **Figure 4D** was extracted and then subjected to qRT-PCR for detection of VP16 RNA levels.


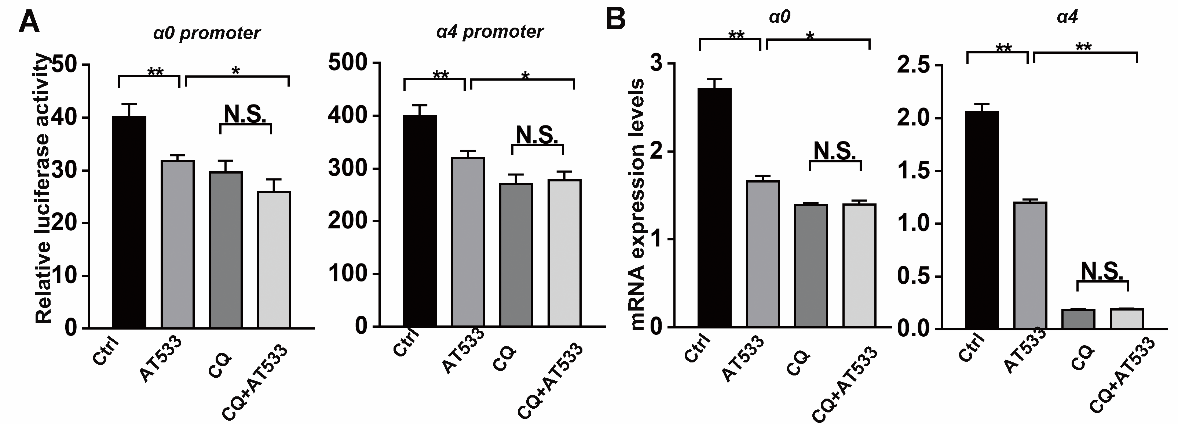


**Figure S5** **A.** Vero cells were transfected with reporter plasmids pGL-*α0* or pGL-*α4* (0.5 μg) for 24 h, treated with AT533 (2 μM) and CQ (50 μM) for 2 h, and then subjected to luciferase activity assays. **B.** Vero cells were infected with HSV-1 (MOI 50) in the presence of AT533 and CQ at 2 h.p.i., and total RNA was extracted for analysis of *α0* and *α4* RNA levels by qRT-PCR (B).
